# A morphological study of schizophrenia with magnetic resonance imaging, advanced analytics, and machine learning

**DOI:** 10.3389/fnins.2022.926426

**Published:** 2022-08-15

**Authors:** Jacob Levman, Maxwell Jennings, Ethan Rouse, Derek Berger, Priya Kabaria, Masahito Nangaku, Iker Gondra, Emi Takahashi

**Affiliations:** ^1^Department of Computer Science, St. Francis Xavier University, Antigonish, NS, Canada; ^2^Center for Clinical Research, Nova Scotia Health Authority - Research, Innovation and Discovery, Halifax, NS, Canada; ^3^Department of Radiology, Massachusetts General Hospital, Athinoula A. Martinos Center for Biomedical Imaging, Harvard Medical School, Massachusetts Institute of Technology, Boston, MA, United States; ^4^Department of Mathematics and Statistics, St. Francis Xavier University, Antigonish, NS, Canada; ^5^Department of Medicine, Boston Children’s Hospital, Harvard Medical School, Boston, MA, United States

**Keywords:** schizophrenia, magnetic resonance imaging, machine learning, depression, correlation analysis

## Abstract

We have performed a morphological analysis of patients with schizophrenia and compared them with healthy controls. Our analysis includes the use of publicly available automated extraction tools to assess regional cortical thickness (inclusive of within region cortical thickness variability) from structural magnetic resonance imaging (MRI), to characterize group-wise abnormalities associated with schizophrenia based on a publicly available dataset. We have also performed a correlation analysis between the automatically extracted biomarkers and a variety of patient clinical variables available. Finally, we also present the results of a machine learning analysis. Results demonstrate regional cortical thickness abnormalities in schizophrenia. We observed a correlation (rho = 0.474) between patients’ depression and the average cortical thickness of the right medial orbitofrontal cortex. Our leading machine learning technology evaluated was the support vector machine with stepwise feature selection, yielding a sensitivity of 92% and a specificity of 74%, based on regional brain measurements, including from the insula, superior frontal, caudate, calcarine sulcus, gyrus rectus, and rostral middle frontal regions. These results imply that advanced analytic techniques combining MRI with automated biomarker extraction can be helpful in characterizing patients with schizophrenia.

## Introduction

Schizophrenia is a mental disorder characterized by hallucinations, delusions, thought disorders and a lack of motivation. Although a number of post-mortem brain studies in the early twentieth century failed to find abnormalities in schizophrenia (e.g., Southard, 1910, 1915; Jacobi and Winkler, 1928; Haug, 1962), the first computer assisted tomography (CT) study succeeded in observing enlarged lateral ventricles (Johnstone et al., 1976). More recently, magnetic resonance imaging (MRI) was introduced in schizophrenia research. MRI-based studies, as well as CT-based studies, confirmed ventricular enlargement, and it has become a consensus that ventricular enlargement is present in first episode patients who are not yet treated by medication (Nordström and Williamson, 2003). The use of MRI has also led to successes in evaluating abnormalities regionally, including investigations of the medial temporal lobe, the prefrontal, orbitofrontal, and parietal lobe in addition to the ventricles (see reviews in Shenton et al., 2001; Kubicki et al., 2005; Haukvik et al., 2013).

The analysis of patients with schizophrenia by MRI examinations has been the subject of many studies. However, existing research has been limited in the populations studied, particularly in terms of the ages of the patients in the study, and in terms of the regions analyzed (Kim et al., 2012; Zalesky et al., 2012; Wu et al., 2015). The use of MRI in schizophrenia research has been further supported by the use of automated measurement tools, such as FreeSurfer (Fischl, 2012). FreeSurfer is a brain imaging analysis technology that aids in identifying and extracting measurements from cortical and non-cortical regions of the brain. It performs volume-based and surface-based analyses, reconstructs accurate models of gray/white matter and pial surfaces, and provides a variety of measurements, such as cortical thicknesses, surface areas, and folding measurements. Studies of schizophrenia combining MRI technology and FreeSurfer have found interdependence between abnormal white matter connections and altered gray matter structure (Liu et al., 2014), reduced hippocampal volume (Arnold et al., 2015; Haukvik et al., 2015; Singh et al., 2018), reduced amygdalar volume (Rich et al., 2016), reduced cerebellar cortical volume (Laidi et al., 2015), thinner cortices (van Erp et al., 2018), and cortical (Yasuda et al., 2020) and subcortical brain volume abnormalities (van Erp et al., 2014) associated with the condition. These findings imply that a variety of abnormalities are present in patients with schizophrenia, and that FreeSurfer technology (Fischl, 2012) is capable of assisting in characterizing regional irregularities potentially associated with the condition.

The application of machine learning to MRI examinations of patients with schizophrenia has been the subject of multiple review articles (de Fillippis et al., 2019; Steardo et al., 2020). Briefly, previous research in the application of machine learning to schizophrenia diagnostics has focused on discriminating schizophrenic patients from those with bipolar disorder (Schnack et al., 2014) and healthy controls (Nieuwenhuis et al., 2012; Iwabuchi et al., 2013; Schnack et al., 2014). Work has also focused on using machine learning to classify childhood-onset schizophrenia (Greenstein et al., 2012). Additional research has focused on differential diagnosis between patients with schizophrenia with and without auditory hallucinations using resting state functional MRI (Chyzhyk et al., 2015), as well as classifying schizophrenic patients into cognitive subtypes (Gould et al., 2014). The combination of MRI and genetic data to improve classification of schizophrenia has also been investigated (Yang et al., 2010). Machine learning (ML) applied to MRI exams of patients with schizophrenia has been the subject of a review article (Veronese et al., 2013) and meta-analysis (Kambeitz et al., 2015). More recently, research has focused on the use of deep learning on structural MRI exams, achieving widely varying performance (area under the curve ranges from 0.71 to 0.90) on unseen datasets (Oh et al., 2020), ML based on whole brain white matter fractional anisotropy (accuracy: 62%) (Mikolas et al., 2018), ML applied to functional MRI (accuracy: 79%) (Gallos et al., 2021), and ML based on voxel-based morphometry on a small dataset (accuracy: 88%) (Lu et al., 2016), as well as a more recent study on a larger cohort (accuracy: 75%) (Salvador et al., 2017). Unfortunately, the differing datasets employed result in variation in the patient populations and image acquisition quality across studies, making direct comparison of widely accepted performance metrics, such as overall accuracy, extremely difficult. Additionally, the ability to explain what a ML model has learned, a critical component for medical applications of artificial intelligence (AI), is extremely limited in emerging methods, such as deep learning.

Since FreeSurfer produces such a large array of anatomical measurements (regional volumes, cortical thicknesses, cortical surface area, surface curvature measurements and more), and machine learning typically involves the multivariate analysis of many feature measurements in order to better inform predictions, we hypothesized that FreeSurfer may play a useful role in statistical machine learning-based diagnostic technology. The combination of FreeSurfer measurements with ML has produced papers presenting their leading ML models based on the random forest (Jo et al., 2019; Yassin et al., 2020), the decision tree (Liang et al., 2019) and the support vector machine (de Pierrefeu et al., 2018), including an approach developed on a small cohort focused on only the amygdaloid and hippocampal regions (Guo et al., 2020), as well as methods that combine FreeSurfer with voxel based morphometry (Schwartz et al., 2019). It should also be noted that ML combined with FreeSurfer has also been applied to the prediction of first episode psychosis (Vieira et al., 2020). FreeSurfer’s validated pipeline produces a wide array of anatomically meaningful and specific measurements, thus ML models employing feature selection and FreeSurfer-based measurement extraction offer inherent improvements in explaining what the model has learned, relative to many of the approaches employed in the literature.

In this study, we hypothesize that a thorough analysis of cortical thickness biomarkers, inclusive of a correlation analysis with clinical characteristics in a schizophrenia population, and a machine learning analysis applied to the many biomarkers available in FreeSurfer (Fischl, 2012), may assist in the characterization of schizophrenia.

## Materials and methods

### Participants, data acquisition and preprocessing

Following approval by BCH’s Institutional Review Board (informed consent was waived due to the lack of risk to participants included in retrospective analyses), the MCIC medical imaging electronic database (Gollub et al., 2013)^1^ was accessed, and all examinations with clinical data available from the MCIC dataset were downloaded for further analysis. Imaging was performed with MRI scanners. The MCIC project was supported by the Department of Energy under Award Number DE-FG02-08ER64581. MCIC is the result of efforts of investigators from the *University of Iowa*, *University of Minnesota*, *University of New Mexico*, and *Massachusetts General Hospital*. Patients were only included if they met the DSM-IV criteria for schizophrenia, schizoaffective disorder or schizophreniform disorder. A major effort was made to only include patients who were antipsychotic drug naïve and were recruited early in the course of their illness. The control participants were selected based on having no history of substance abuse or psychiatric illness, and were matched to the patient cohort for age, sex, and parental education (Gollub et al., 2013). Data used in the preparation of this work were obtained from the Mind Clinical Imaging Consortium database (see text footnote 1) through the Mind Research Network.^2^ The detailed protocol descriptions available are in the literature (Gollub et al., 2013).

Each T1 structural examination was processed with FreeSurfer (Fischl, 2012), using the recon-all command which aligns the input examination to all available atlases. All atlases that have cortical thickness measurements were included for further analysis. These combined atlases include definitions of 331 cortical regions in the brain, divided into both the left and right hemispheres. Each FreeSurfer output T1 structural examination was displayed with label map overlays and visually inspected for regional segmentation quality. If FreeSurfer results were observed to substantially fail, they were excluded from this analysis [i.e., FreeSurfer regions-of-interest (ROIs) that do not align to the MRI and examinations where major problems were observed with an ROI such as a cerebellar segmentation extending far beyond the extent of the cerebellum]. This resulted in a collection of 174 MRI examinations that passed quality control (from 213 examinations publicly accessed), including 99 examinations of patients with schizophrenia, and 75 examinations of healthy control participants.

### Statistical analysis

This study included the acquisition of 662 regionally distributed cortical thickness measurements per imaging examination (mean and standard deviation of within region thicknesses across both left and right hemispheres), as extracted by FreeSurfer’s recon-all command, which processes the participant’s examination with all available atlases (Fischl, 2012). This included extracting measurements of the average and the standard deviation of within-region cortical thicknesses for each supported gray matter region. Cohen’s *d* statistic (positive/negative values indicate a higher/lower average value in the schizophrenic population relative to the neurotypical population) and a p-value based on the standard *t*-test (Student, 1908) for two groups of samples were computed to assist in the assessment of group-wise differences between our two populations. The p-value was selected as an established method to demonstrate that it is unlikely that our findings were the result of random chance, Cohen’s *d* statistic was selected as it is the most established method to assess effect sizes. This yielded a total of *m* = 662 group-wise comparisons, yielding a Bonferroni corrected threshold for achieving statistical significance of *p* < 0.05/*m* = 7.55e^–5^.

In order to confirm that the findings reported are the result of group-wise differences between the schizophrenic and neurotypical participants, a statistical model was constructed based on multivariate regression (using MATLAB’s mvregress function), adjusting each measurement in order to control for group-wise differences in age, gender and imaging site. This model was used to adjust each cortical thickness (mean and standard deviation) measurement, in order to evaluate whether group-wise differences between our pathological and typically developing populations are the result of age, gender or imaging site effects.

A correlation analysis was performed to assess possible relationships between cortical thickness measurements extracted from MRI examinations and patient clinical variables from the schizophrenia population. In the MCIC dataset, a variety of clinical variables are available for each pathological patient, including, age, gender, parental education levels, a variety of neurological test results, etc. A detailed description of all of the variables available is provided in the literature (Gollub et al., 2013). Each of 662 cortical thickness measurements were compared with each of the 90 clinical variables available for our schizophrenic population with a correlation analysis, computing Pearson’s correlation coefficient and an associated p-value. This resulted in *m* = 662 × 90 = 59,580 comparisons, yielding a Bonferroni corrected threshold for achieving statistical significance of *p* < 0.05/*m* = 8.39e^–7^.

### Machine learning analysis

Five machine learning algorithms are compared in this analysis, the support vector machine (SVM), the decision tree (DT), the random forest (RF), bagged logistic regression (BL), and an artificial neural network (ANN). All examinations that passed quality control were included in the machine learning analysis. All 4,784 feature measurements produced by FreeSurfer’s recon-all command were included as potential features to be selected by the machine learning algorithms. Hyperparameter tuning was employed with each algorithm in order to optimize classifier performance using Optuna Bayesian optimization, which performed extremely well in a recent public competition (Turner et al., 2021). The parameters subjected to Optuna Bayesian optimization for each ML method are as follows: RF – the number of estimators/trees, the criterion (gini vs. entropy), the tree depth, and whether or not to bootstrap; SVM – C the regularization parameter; DT – criterion (gini vs. entropy), tree depth, whether splits are random vs. best; BL – number of estimators, feature down sampling, whether to bootstrap or not; ANN – number of layers, layer size, regularization, batch size, learning rate, learning rate decay. Each of these algorithms were combined with each of three feature selection techniques: ranking features based on Cohen’s *d* statistic, Principal Components Analysis (PCA) and stepwise feature selection. The number of features included for each of the feature selection methods was set to each of 10, 50, and 100. Each combination of machine learning algorithm and feature selection technique was evaluated as part of a K-Fold cross validation, with *K* = 5, K-Fold cross validation with *K* = 10, and Monte-Carlo style bootstrapping, ensuring a large share of examinations were available for model training with held out samples used for testing/model validation. This validation was repeated 100 times. In each run of the validation, the overall accuracy (OA) is computed alongside the area under the receiver operating characteristic curve (AUC), the sensitivity and the specificity. Summary statistics (mean and standard deviation) are computed across validation runs. All machine learning, validation and statistical analyses were performed in python. Public domain software has been made available at https://github.com/stfxecutables/df-analyze to facilitate other researchers to (1) reproduce the findings of this study, and (2) to facilitate the application of the ML methods used herein to any given data frame (samples in rows, feature measurements in columns), helping make the addition of Optuna hypertuned ML easy in future studies.

## Results

### Cortical thickness analysis

Many regions demonstrated group-wise differences between participants with schizophrenia and healthy controls. Decreases in average cortical thicknesses with the largest effect sizes were observed in the superior temporal, temporal pole, middle temporal, and fusiform regions. These findings are summarized in Table 1, demonstrating consistently decreased cortical thicknesses in patients with schizophrenia. All findings provided in Table 1 represent raw (not multivariable regression adjusted) effect size calculations, all of which had statistically significant associated raw p-values, as well as statistically significant multivariable adjusted p-values computed with the results of the multivariable regression analysis that controls for the effect of age, gender, and imaging site.

**TABLE 1:** Effect sizes for regions exhibiting statistically significant group-wise differences in regional average thickness.

Regional measurement	Cohen’s *d*
Left superior temporal average thickness	−0.728
Right temporal pole average thickness	−0.717
Left middle temporal average thickness	−0.712
Right fusiform average thickness	−0.678
Left superior segment of the circular sulcus of the insula average thickness	−0.672
Left inferior temporal average thickness	−0.654
Left hemisphere average thickness	−0.654
Left lateral superior temporal gyrus average thickness	−0.650
Left temporal pole average thickness	−0.645
Left middle occipital gyrus average thickness	−0.636
Right superior temporal average thickness	−0.634
Right anterior transverse collateral sulcus average thickness	−0.631
Left superior temporal sulcus average thickness	−0.630
Right anterior segment of the circular sulcus of the insula average thickness	−0.625
Left inferior segment of the circular sulcus of the insula average thickness	−0.618
Right inferior temporal average thickness	−0.618
Right pars orbitalis average thickness	−0.600
Right middle temporal average thickness	−0.593
Left fusiform average thickness	−0.592
Left Brodmann’s area 45 average thickness	−0.592

Negative Cohen’s d values indicate that the schizophrenic population have a lower average measured value as compared with the healthy patients.

Unlike other cortical thickness studies, which generally focus on average cortical thicknesses, our study has also included intra-regional cortical thickness variability measurements in the form of the standard deviation. Results indicate a common tendency for schizophrenic patients to exhibit increased cortical thickness variability across many sub-regions of the brain. However, the findings are based on small to medium effect sizes that do not reach our stringent standard for statistical significance. The largest Cohen’s *d* statistics associated with the abnormalities of intra-regional cortical thickness variability was found in the left rostral anterior cingulate (*d* = 0.44), the left pars orbitalis (*d* = 0.38), the triangular part of the left inferior frontal gyrus (*d* = 0.34) and the left rostral middle frontal region (*d* = 0.33).

### Cortical thickness to clinical variable correlation analysis

The correlation analysis considered the schizophrenia population whose examinations passed quality control, thus this analysis helps assess possible relations between automated extractable brain biomarkers and clinical variables in a schizophrenic population. The correlation analysis yielded 156 biomarker measurement and clinical variable pairs that exceeded the Bonferroni correction, or just 0.26% of all pairings between automatically extracted biomarkers and clinical variables. The most common statistically significant correlations established were negative correlations between patient age and average cortical thicknesses (78/156 statistically significant measurements) of a wide variety of regions, confirming known cortical thinning that progresses with age (e.g., Salat et al., 2004), as well as confirming this effect specifically in schizophrenia (Kubota et al., 2011). The second most common statistically significant correlations were found to be negative correlations between the amount of time a patient takes to complete a clinical assessment task and regional cortical thicknesses (57/156 statistically significant measurements), a finding that may also be age-dependent. The third most common statistically significant correlations were found to be negative correlations between illness duration and regional average cortical thicknesses (17/156 statistically significant measurements), another finding that might be a product of age-dependency, as older patients naturally have thinner cortices and older patients, on average, have had schizophrenia for longer periods of time. We observed a negative correlation between the thickness of the left precentral cortex and the number of errors patients make (Error Score clinical variable; rho = −0.482). Finally, a positive correlation was observed between the Calgary Depression Scale (Addington et al., 1993) total score and the right medial orbitofrontal average cortical thickness (rho = 0.474).

### Machine learning analysis

In Table 2, the mean and standard deviation of OA and AUC, as well as sensitivity and specificity, are provided for each of our top performing combination of machine learning methods and feature selection techniques. Results in Table 2 demonstrate considerable variability in machine learning performance depending on the underlying technology as well as the feature selection technique chosen. The best performing model was the support vector machine (SVM) with stepwise feature selection (OA: mean 84%, standard deviation 4%) limited to 50 features. Similar results were obtained from the SVM with stepwise feature selection with only 10 features (OA: mean 83%, standard deviation 5%). These leading models both based their predictions on the variability of the thickness of the left calcarine sulcus, the volume of the left gyrus rectus, the number of vertices on the left superior frontal gyrus, the average thickness of the left rostral middle frontal region, the signal intensity of the right insula, right superior frontal gray matter, and right caudate, the left hemisphere surface integral, and the irregularity of the left hemisphere’s surface reconstruction (in terms of defect holes). All results in Table 1 report validation findings for K-Fold cross validation (*K* = 5) repeated 100 times reporting on the average and standard deviation of each performance metric. Overall, stepwise feature selection was consistently the best performing feature selection method available.

**TABLE 2:** Comparative results of 5 ML algorithms and three feature selection (FS) techniques in terms of overall accuracy (OA), area under the receiver operating characteristic curve (AUC), sensitivity, and specificity.

FS: stepwise	SVM	DT	RF	BL	ANN
OA mean (Std Dev)	0.84 (0.04)	0.75 (0.07)	0.84 (0.06)	0.83 (0.05)	0.74 (0.08)
AUC mean (Std Dev)	0.83 (0.04)	0.75 (0.08)	0.84 (0.05)	0.83 (0.06)	0.74 (0.09)
Sensitivity mean (Std Dev)	0.92 (0.08)	0.79 (0.05)	0.84 (0.11)	0.85 (0.04)	0.79 (0.13)
Specificity mean (Std Dev)	0.74 (0.09)	0.70 (0.12)	0.85 (0.07)	0.81 (0.13)	0.69 (0.22)
# of features	50	100	50	100	50
**FS: PCA**	**SVM**	**DT**	**RF**	**BL**	**ANN**

OA mean (Std Dev)	0.64 (0.11)	0.62 (0.05)	0.63 (0.05)	0.63 (0.07)	0.59 (0.08)
AUC mean (Std Dev)	0.63 (0.11)	0.62 (0.05)	0.62 (0.05)	0.61 (0.08)	0.57 (0.08)
Sensitivity mean (Std Dev)	0.70 (0.16)	0.66 (0.08)	0.68 (0.10)	0.77 (0.04)	0.71 (0.22)
Specificity mean (Std Dev)	0.56 (0.18)	0.58 (0.11)	0.57 (0.12)	0.44 (0.15)	0.44 (0.25)
# of features	10	10	10	50	100
**FS: *D***	**SVM**	**DT**	**RF**	**BL**	**ANN**

OA mean (Std Dev)	0.64 (0.12)	0.62 (0.07)	0.64 (0.06)	0.67 (0.05)	0.60 (0.05)
AUC mean (Std Dev)	0.63 (0.12)	0.61 (0.08)	0.64 (0.06)	0.67 (0.06)	0.58 (0.07)
Sensitivity mean (Std Dev)	0.70 (0.10)	0.71 (0.08)	0.67 (0.11)	0.69 (0.07)	0.73 (0.19)
Specificity mean (Std Dev)	0.57 (0.15)	0.51 (0.15)	0.61 (0.16)	0.65 (0.11)	0.43 (0.29)
# of features	100	50	50	50	10

The feature selection (FS) method is listed in the top left. The symbol # denotes the word number.

## Discussion

We performed a retrospective analysis of a public schizophrenia dataset including a cortical thickness analysis and a correlation analysis. Our findings from the cortical thickness analysis confirm known average group-wise cortical thinning in schizophrenia (van Erp et al., 2018) in the superior temporal cortex (Schultz et al., 2010; van Haren et al., 2011), middle temporal gyrus (Cui et al., 2018), right fusiform gyrus (Goghari et al., 2015), inferior temporal cortex (Buchy et al., 2011), and thinning of the right temporal pole, which was observed in patients at high risk for schizophrenia (Li et al., 2016). We observed cortical thinning of the left temporal pole, which has previously been reported to be correlated with processing speed (Hartberg et al., 2011). Additionally, we have observed cortical thinning in the left middle occipital gyrus, which has been reported to be linked with the verbal intelligence quotient (IQ) in schizophrenia (Hartberg et al., 2010). We were also able to confirm cortical thinning in Brodmann’s area 45 (Narayan et al., 2007). Various studies have reported abnormalities of the insula (Shepherd et al., 2012), including abnormalities of insular cortical thickness (Song et al., 2015; Emami et al., 2016), which this study has confirmed alongside reporting thinning of the insular sulcus.

In this study, the majority of the statistically significant correlations between clinical variables and structural cortical thickness measurements were observed between patient age and regional cortical thicknesses that were highly negative correlations. These findings are expected, as it is well known that cortical thinning (reductions in average cortical thickness) occurs naturally with age (Fjell et al., 2009; Levman et al., 2017), that schizophrenia populations exhibit increased cortical thinning relative to neurotypical controls (Kubota et al., 2011; Nenadic et al., 2015), and, specifically, negative correlations have been reported between temporal pole thickness and age in schizophrenia (van Erp et al., 2018). Furthermore, we observed many (17/156) statistically significant negative correlations between illness duration and regional average cortical thicknesses, a finding in agreement with previously completed research (van Erp et al., 2018). Of potentially more interest, findings include a positive correlation between the Calgary Depression Scale For Schizophrenia Total Score (Addington et al., 1993) and the average cortical thickness of the right medial orbitofrontal region, which has been implicated in depression outside of a schizophrenic population (Drevets, 2007; Cheng et al., 2016). These results are supportive of previous literature findings of observed orbitofrontal abnormalities in schizophrenia (Waltz and Gold, 2007; Kanahara et al., 2013), and imply that the right medial orbitofrontal region might be directly implicated in the experience of depression among patients with schizophrenia. We observed a negative correlation between the thickness of the left precentral region and the number of errors patients make (Error Score clinical variable; rho = −0.482), which may be supportive of reported associations of the precentral gyrus with No-Go errors (Stevens et al., 2009) and reported functional MRI-based activation-behavior correlations in the precentral gyrus in Go trials (Zhang et al., 2019), both in control populations.

Multivariable regression models were included to assess the possible confounding effects of age, gender and imaging site. Results indicated that our primary findings remained statistically significant when controlling for these factors. We elected to present our raw (unadjusted) results rather than our multivariable regression model results for ease of comparison and reproducibility with future studies, and because it has been previously demonstrated that the use of multivariable regression in neuroscience is associated with the introduction of error, and this was specifically demonstrated on this same schizophrenia dataset (Levman et al., 2021).

We performed a comparative analysis of five machine learning methods and three feature selection methods combined with Optuna hyperparameter tuning toward the automated diagnosis of schizophrenia from T1 structural MRI examinations. The leading model was a support vector machine employing stepwise feature selection, yielding an accuracy of 84%. This leading model based its predictions on a variety of identified abnormalities implicating the right insula, right superior frontal gray matter, right caudate, left calcarine sulcus, left gyrus rectus, left superior frontal gyrus, the left rostral middle frontal region, and whole left hemisphere surface measurements in schizophrenia. Many of these brain regions have been previously identified as being involved in schizophrenia, including the insula (Wylie and Tregellas, 2010), superior frontal gyrus (Gao et al., 2015; Vogel et al., 2016), caudate (Ebdrup et al., 2010; Williams, 2016), calcarine sulcus (Sulejmanpašić et al., 2016), gyrus rectus (Kim et al., 2017), and the rostral middle frontal region (Kikinis et al., 2010). The results of our ML analysis directly support these historical literature findings. Furthermore, these historical findings lend support to the potential reliability of the leading machine learning model developed as part of the methods from this analysis.

Deep learning is a popular approach to machine learning applications in medical imaging. Research has focused on the use of deep learning on structural MRI exams for the prediction of schizophrenia, achieving widely varying performance (area under the curve ranges from 0.71 to 0.90) on unseen datasets (Oh et al., 2020), implying that current deep learning technologies may not meet the high reliability standards expected in medical applications. This is further supported by recent findings applying deep learning to schizophrenia diagnostics, which achieved accuracy of 70% at best (Vieira et al., 2020).

Accuracy of reported ML models that rely on more traditional techniques (not deep learning) varies considerably, with techniques reporting accuracies of 62% for ML based on whole brain white matter fractional anisotropy (Mikolas et al., 2018), 79% for ML applied to functional MRI (Gallos et al., 2021), 88% for ML based on voxel-based morphometry on a small dataset (Lu et al., 2016), as well as 75% accuracy on a larger cohort of participants (Salvador et al., 2017). Automated feature extraction technologies, like FreeSurfer (Fischl, 2012), provide detailed feature measurements that are more informative relative to that obtainable from deep learning and voxel based morphometry (VBM) based approaches. The application of ML to FreeSurfer measurements has been the subject of existing work, which has produced papers presenting their leading ML models with accuracies of 76 and 69%, respectively, based on the random forest (Jo et al., 2019; Yassin et al., 2020), 72% based on the support vector machine (de Pierrefeu et al., 2018), 82% based only on the amygdaloid and hippocampal regions in a small cohort of study participants (Guo et al., 2020), and 76% accuracy from combining FreeSurfer with VBM (Schwartz et al., 2019). Unfortunately, the differing datasets employed result in variation in the patient populations and image acquisition quality across studies, making direct comparison of widely accepted performance metrics, such as overall accuracy, extremely difficult. Thus, directly comparing two OA values from differing studies should be done with great caution. However, it is noted that our approach, reporting OA of 84%, exhibits strong performance based on FreeSurfer (Fischl, 2012) extracted measurements, and the closest performing model (Guo et al., 2020), with 82% OA, was based on measurements extracted from just the amygdaloid and hippocampal regions and was based on a small patient population. Our approach achieves the results outlined with biomarkers extracted from many regions, including the insula, superior frontal region, etc. FreeSurfer based methods have considerable improved potential to inform clinicians of the brain features that contribute to a patient being diagnosed with schizophrenia.

From a technical perspective, the best performing ML technique investigated was the support vector machine (SVM) combined with stepwise feature selection. The SVM is a classical ML approach that attempts to minimize error on unseen samples, which is a statistical sampling dependent approach that is capable of providing substantial reliability improvements over many ML techniques. Additionally, the SVM operates by defining a boundary between predictions based on training samples located near the boundary itself, providing potentially statistically robust solutions to the discrimination/prediction problem. Artificial neural networks (ANNs) perform feature weighting and are based on extensive random initialization, which potentially contributes to variability in performance across validation runs. The decision tree and the random forest do not weight their individual features, however, some features are prioritized over others based on their respective positions in the resultant decision trees associated with these methods. The support vector machine does not perform feature weighting, however, its robust sample down sampling strategy can sometimes result in a minimization of the effects of nuisance features. Employing feature selection with limits on the number of features helps to ensure that models do not rely on excessive numbers of spurious features to inform prediction, thus potentially contributing to improvements in ML reliability.

Stepwise feature selection was the best performing of the three feature selection methods considered in this analysis. Stepwise feature selection involves repeatedly adding feature measurements to the machine learning model based on which measurement is anticipated to provide the most improvement to the accuracy of the resultant technology. Unfortunately, stepwise feature selection is by far the slowest method of those evaluated. Stepwise feature selection does allow the user to select the number of features included in the ML model, which when set appropriately low, can assist in the creation of reliable artificial intelligence (AI) technologies. It is noteworthy that stepwise feature selection outperformed the other two feature selection methods (PCA and ranking with Cohen’s *d* statistic) for all five machine learning algorithms assessed. PCA tries to rotate the high dimensional data space in order to identify salient projections that act as rotated features. It is a popular dimensionality reduction technique that can be used to reduce the number of features by keeping only those rotated measurements that allow us to retain as much variance in the dataset as possible, and thus hopefully retains as much discriminatory information as possible. Ideally, the technique will keep rotated features that result in large interclass distances and small intraclass variance in the feature space. It is also desirable to assess correlations between features, as is done in PCA, so that any redundant information can be removed. The basic idea in PCA is to find the linearly transformed components that can explain the maximum possible amount of variance in the dataset. Unfortunately, the input dataspace is very large in terms of input features and sparsely populated, which helps explain why the technique is not functioning reliably. Cohen’s *d* statistic represents an established method for assessing a feature measurement’s effect size. Ranking our features with Cohen’s *d* statistic and providing the leading set of features to the machine learning algorithm produced results of intermediate quality in this application.

The limitations of the study include that disease sub-status was not included as a variable in the analyses, thus no group-wise comparisons nor statistical models were constructed to control for issues such as whether a given patient had experienced chronic or first episode schizophrenia, respectively. Additional limitations include that the study was performed on a modest sized publicly available dataset. Strengths of the study include a correlation analysis, as well as a machine learning analysis, and open source machine learning code to facilitate the reproducibility of our study findings.

In conclusion, our analysis included the observation of correlations between cortical thickness of the right medial orbitofrontal cortex and patient depression in schizophrenia, as well as provided extensive sets of results from machine learning, indicating good performance for predicting the condition from computer code that we have made publicly available. Future work is needed to further optimize the performance of machine learning in this application. Future work will investigate the extent of improvements to machine learning diagnostics attainable by dramatically increasing the sample size and investigating the use of deep learning based convolutional neural networks. It is hoped that as new technologies in explainable deep learning become available, that methods such as those presented in this paper can assist with comparative validation between models to ensure sensible behavior from emergent deep learning technologies. All underlying features and trained models need to be subjected to rigorous validation on independently acquired datasets. We will also extend the analysis to include additional MRI modalities, such as tractography and functional MRI, as well as expanding our analyses to larger datasets. It is hoped that reliable ML models based on validated FreeSurfer measurement extraction technology can act as comparative models to help validate emerging explainable deep learning models as part of future work as well. Future work will also perform additional analyses to control for the effects of disease sub-status, which was not included as a variable in our analyses, in order to assess the effects associated with whether a given patient had experienced chronic or first episode schizophrenia, respectively. It is hoped that these research avenues will assist toward better understanding schizophrenia as well as improved characterization, diagnosis and classification of the disorder.

## Data availability statement

The data relied upon in this study is publicly available. Data used in the preparation of this work were obtained from the Mind Clinical Imaging Consortium database through the Mind Research Network: https://coins.trendscenter.org/.

## Ethics statement

The studies involving human participants were reviewed and approved by Boston Children’s Hospital REB. Note this is a publicly available dataset so no REB permission is required. The patients/participants provided their written informed consent to participate in this study.

## Author contributions

JL: conceived the study. JL, ET, and IG: study supervision and feedback on and editing the manuscript. MJ, ER, and DB: software development. JL, PK, and MN: authoring the manuscript. All authors contributed to the article and approved the submitted version.

## Footnotes

1
https://coins.trendscenter.org/

2
www.mrn.org
